# SNP-set analysis replicates acute lung injury genetic risk factors

**DOI:** 10.1186/1471-2350-13-52

**Published:** 2012-06-28

**Authors:** Nuala J Meyer, Zhongyin John Daye, Melanie Rushefski, Richard Aplenc, Paul N Lanken, Michael GS Shashaty, Jason D Christie, Rui Feng

**Affiliations:** 1 Department of Medicine: Pulmonary, Allergy, and Critical Care Division, Perelman School of Medicine University of Pennsylvania, 3600 Spruce Street, 874 Maloney, Philadelphia, PA 19104, USA; 2 Department of Biostatistics and Epidemiology, Center for Clinical Epidemiology and Biostatistics, Perelman School of Medicine University of Pennsylvania, Philadelphia, PA, USA; 3 Division of Hematology – Oncology, Children’s Hospital of Philadelphia, Philadelphia, PA, USA

**Keywords:** Genetic association study, Acute respiratory distress syndrome, Kernel machine regression

## Abstract

**Background:**

We used a gene – based replication strategy to test the reproducibility of prior acute lung injury (ALI) candidate gene associations.

**Methods:**

We phenotyped 474 patients from a prospective severe trauma cohort study for ALI. Genomic DNA from subjects’ blood was genotyped using the IBC chip, a multiplex single nucleotide polymorphism (SNP) array. Results were filtered for 25 candidate genes selected using prespecified literature search criteria and present on the IBC platform. For each gene, we grouped SNPs according to haplotype blocks and tested the joint effect of all SNPs on susceptibility to ALI using the SNP-set kernel association test. Results were compared to single SNP analysis of the candidate SNPs. Analyses were separate for genetically determined ancestry (African or European).

**Results:**

We identified 4 genes in African ancestry and 2 in European ancestry trauma subjects which replicated their associations with ALI. Ours is the first replication of IL6, IL10, IRAK3, and VEGFA associations in non-European populations with ALI. Only one gene – VEGFA – demonstrated association with ALI in both ancestries, with distinct haplotype blocks in each ancestry driving the association. We also report the association between trauma-associated ALI and NFKBIA in European ancestry subjects.

**Conclusions:**

Prior ALI genetic associations are reproducible and replicate in a trauma cohort. Kernel - based SNP-set analysis is a more powerful method to detect ALI association than single SNP analysis, and thus may be more useful for replication testing. Further, gene-based replication can extend candidate gene associations to diverse ethnicities.

## Background

Acute lung injury (ALI) is a syndrome of flooded alveolar spaces, severe hypoxemia, and acute respiratory failure [1] which afflicts approximately 190,000 individuals in the United States each year [2]. There is widespread interest to identify genetic risk factors contributing to ALI susceptibility [3-6], because ALI susceptibility is incompletely explained by clinical risk factors and the morbidity and mortality associated with ALI are substantial [2,7]. While it is difficult to estimate heritability for ALI given its necessity for a severe environmental insult such as severe injury or exposure to a ventilator, there is strong evidence for a heritable basis underlying individual response to injury and inflammation [8-11]. Offspring whose parents died prematurely from infection sustained a 6-fold higher risk of themselves dying of infectious causes, a heritability much stronger than for vascular diseases or cancer [12]. Furthermore, as there exists no proven pharmacologic therapy for patients with ALI, it may be that the discovery of individualized risk factors for ALI could advance the development of personalized therapy for subjects with or at risk for ALI. Variants in 29 genes have now been implicated as risk factors contributing to ALI susceptibility or outcome [3,5,13-16], though only 10 of these associations have been replicated in more than one population.

Traditional approaches to detect genetic risk variants identify single nucleotide polymorphisms, or SNPs, that identify a consistent association with the phenotype of interest. As high-throughput genotyping technologies have become accessible, it is now possible to test hundreds and thousands of SNPs simultaneously, allowing for maximal efficiency. However, to account for the increasing probability of detecting false positive associations with multiple testing, single SNP association tests rely upon stringent statistical thresholds to claim significance. This approach, which corrects for the number of SNPs tested, is robust to statistical review but has several shortcomings. The single SNP method fails to account for the relationship between SNPs which may travel together, known as linkage disequilibrium (LD) blocks or haplotypes; it accords no weight to previously hypothesized candidate genes with *in vitro* or animal evidence to support a pathogenic role in the phenotype of interest; and it may discard as non-significant all but the most extreme associations or the largest effect sizes. Further, by ranking SNPs on the basis of their parametric *p* values, obtained by regressing the phenotype onto each SNP, many of the very top ranked SNPs may be false positives which cannot be replicated [17].

An alternative to the individual SNP approach is to group SNPs into haplotype blocks – the subset of SNPs which tend to be inherited together – and to test association for all members of the block jointly. This strategy allows multiple correlated SNPs that are members of a gene product to inform the association. By testing LD blocks rather than individual SNPs, fewer hypotheses are tested, and the statistical threshold for significance can be relaxed. Further, as opposed to individual SNP analyses which rely on the genotyped SNP acting as a surrogate for the causal SNP, the LD block as a whole may perform as a more correlated marker for the untyped causal SNP. In addition, SNP-set analysis can potentially evaluate within-block epistatic effects, or interactions between groups of SNPs on the phenotype. Epistasis is classically understood as the effect at one locus altering the effect of another allele on the phenotype being studied. Statistically, it is detected by finding that the 2-locus genotype frequency varies with respect to phenotype more than would be predicted by summing the allelic effects on the phenotype at each locus [18]. Several complex traits such as non-insulin dependent diabetes and precocious breast cancer have demonstrated significant gene by gene, or epistatic, influences [17,19,20]. By detecting minor allele sharing and SNP-SNP interactions with a phenotype, SNP-set analysis may be a powerful tool to detect meaningful associations when individual SNP associations are modest [17].

We hypothesized that a candidate gene SNP array, designed to capture dense genotyping of approximately 2000 genes strongly hypothesized to play a role in vascular, inflammatory, or metabolic processes, would be particularly informative for SNP-set analysis of ALI risk given its fine resolution of linkage disequilibrium blocks for multiple ancestries [21]. We tested whether the SNP-set method would replicate any previously reported association with ALI candidate genes that were covered by the genotyping platform, as replication is essential to refine the genetic signal as well as the endophenotype, or specific population at risk [22]. Further, we used SNP-set analysis to perform the first large scale replication study of ALI genetic risk factors in African American subjects. Gene – based analytic methods have been proposed as a preferred technique to test previous genetic findings in populations with distinct ancestral structure [23].

## Methods

### Study population

**Figure 1:**
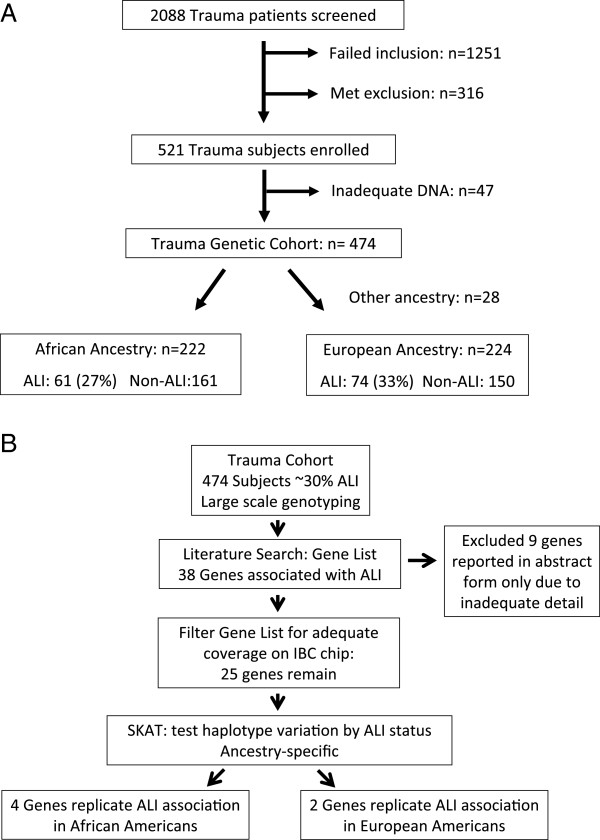
**A: Study population.** All trauma patients were screened. Eligible subjects were severely injured, with an injury severity score (ISS) ≥ 16, and were admitted to the intensive care unit (ICU). Exclusion criteria included isolated head injury and discharge or death within 24 h. Subjects with adequate DNA were genotyped with the IBC SNP array and were classified by genetic ancestry, African (AA) or European (EA), to create haplotypes for ALI candidate genes. **B: Overview of study design and results.** Large scale genotyping data was available for 474 subjects in a trauma cohort. A literature search using the terms “acute lung injury polymorphism,” “acute respiratory distress polymorphism,” or “genetic association lung injury,” limited to human species, was performed in May 2011 and returned 38 genes. Nine genes (IL1RN, ORMDL3, THMD, DARC, DIO2, PRKAG2, ISG15, VWF, and BVES) were cited in abstract form only, and were excluded due to lack of detail about the population or genetic variant studied. Of the 29 remaining genes (Table 2), 4 were not covered by the IBC platform. The remaining 25 genes underwent haplotype imputation for each ancestry and the SNP-set kernel association test was implemented for 3 kernel functions: linear, identical by state, and quadratic. Four genes in subjects of African ancestry and 2 genes in subjects of European ancestry replicated their association with ALI in this trauma cohort.

Subjects were consecutive critically ill trauma patients enrolled in a prospective cohort study of acute lung injury following trauma at the Hospital of the University of Pennsylvania. Patients were eligible if they were transported to the emergency department (ED) following trauma, demonstrated an injury severity score (ISS) ≥ 16, and were admitted to the surgical intensive care unit. Exclusion criteria included isolated head injury, pediatric status, or death or discharge within 24 hours of ED arrival. Further details regarding this cohort have been published [13,24,25] and are depicted in Figure 1A. This study was performed with approval of the University of Pennsylvania Institutional Review Board and was granted waiver of informed consent in accordance with federal and institutional guidelines given its minimal risk (use of residual blood after clinical laboratory use) and to maintain a cohort free of selection bias for critically ill trauma patients [24].

### ALI phenotype

Determination of ALI status was made for 5 days post-trauma in accordance with the American European consensus conference definition [26] for intubated and mechanically ventilated patients. All chest radiographs procured for clinical care during the 5 days post-trauma were interpreted by 2 physicians and adjudicated in the case of disagreement as described [24,25].

### Candidate gene selection

We developed a list of ALI candidate genes based on 2 recent published reviews [3,5] supplemented by individual PubMed ( http://www.ncbi.nlm.nih.gov/pubmed) searches using the terms “acute lung injury polymorphism,” “acute respiratory distress polymorphism,” or “genetic association lung injury,” limited to human species, in May 2011. The PubMed searches were manually curated and candidate genes included if the following criteria were met: 1) the study was a human case control or cohort design; 2) the phenotype of interest was ALI or ARDS by consensus criteria [26], acute respiratory failure requiring intubation in an ALI at-risk population, or death following ALI; and 3) there was a statistically significant association for a variant of that gene in one or more population(s). We did not include abstract publications due to inadequate detail about the genotypes and populations tested. The candidate gene list was filtered for genes that were genotyped to capture at least 50% of the global genetic diversity by the genotyping platform (Figure 1B).

### Genotyping and determination of genetic ancestry

Residual blood samples were obtained after clinical lab use and DNA was extracted from whole blood using the Qiagen Qiamp isolation kit (Qiagen™, Valencia, CA). ALI case and non-case DNA was plated together on 96-well plates, with lab personnel unaware of phenotype designation, and genotyped using the Illumina – Broad – CARe consortium (IBC) designed custom SNP array (Illumina™, San Diego CA), henceforth referred to as the IBC chip [21]. The IBC chip was designed to assay SNPs in approximately 2000 genes strongly hypothesized to play roles in vascular, inflammatory, or metabolic phenotypes specific to lung and cardiovascular diseases, and assesses approximately 50,000 SNPs. We filtered results for SNPs annotated by the CARe consortium to previously reported ALI candidate genes using the literature search methodology described above. We excluded SNPs with missing rate in total population larger than 5%, SNPs with significant departure from Hardy-Weinberg equilibrium (HWE) in non-ALI subjects (p-value <10^−4^) or SNPs with minor allele frequency (MAF) less than 5% in non-ALI subjects. For each haplotype block, individuals with absent genotyping calls for a SNP within the block were also excluded. 

Genetic ancestry was determined using multidimensional scaling (MDS) analysis using all markers on the IBC chip as previously described [13,27]. This yielded 2 dominant ancestral groups, European and African, and then MDS was repeated within each ancestry to remove outliers and to provide principal components for use in adjustment for population stratification. Subsequent analyses were performed separately by genetically determined ancestry.

### Haplotype determination and assignment of SNP-set

For each population – European ancestry (EA) and African ancestry (AA) – haplotype blocks were initially determined by the solid spine method and haplotype frequencies were estimated using the standard expectation maximization algorithm, both implemented in Haploview [28,29]. Small blocks were modified customarily to include at least three SNPs to allow potential within–block SNP interaction for kernel testing.

### SNP-based association testing

For each SNP in a candidate gene, an additive model of genetic risk was assumed and the association was tested using logistic regression, adjusting for 5 clinical covariates: age, injury severity score (ISS), acute physiology and chronic health evaluation (APACHE) III score modified to remove arterial oxygenation information, blunt trauma, and the number of units of blood transfused in the first 24 h post-trauma [24]. The single SNP results of this population have previously been published and reported [13,30] and are used in this publication only as a contrast to the SNP-set kernel association test.

### Multilocus association testing

Using the haplotypes constructed as described above, we tested the joint effect of all SNPs within a haplotype block using the SNP-set function of the sequence kernel association test (SKAT) [17]. This test uses a kernel-machine framework to semi-parametrically model and subsequently test the effects of multiple SNPs grouped into a haplotype. Kernel machine regression allows for either linear or nonlinear relationships between SNPs and phenotype while adjusting for additional covariate effects, measuring the similarity between individuals on the basis of the genotypes of the SNPs in the SNP set. Various kernels can be employed to model different relationships among SNPs within a SNP set, and between a SNP set and the phenotype. We used *i*dentical-*b*y-*s*tate (IBS) and quadratic kernels, as defined below in equations (1 – 3), to allow the incorporation of complex and epistatic effects among SNPs in a set. The IBS kernel incorporates information on the number of minor alleles shared among individuals. The quadratic kernel has the additional feature of incorporating all two-way interactions and quadratic main effects of the SNP set on the association with ALI; SNP – SNP interactions with both consonant and opposing directions on ALI outcome are detected. For each kernel method, results were adjusted for the clinical covariates age, ISS, blunt trauma, modified APACHE score, and amount of blood transfused in the initial 24 h post-trauma [24]. The results from linear, IBS, and quadratic kernels are contrasted with the individual SNP analyses for each candidate gene. A *p* value of 0.05 was considered significant without adjusting for multiple comparisons, as each of the genes tested has previously been reported to associate with ALI.

The SNP-set function of SKAT can be elaborated as follows. For an individual *i*, let $x_{\mathrm{il}},\ldots ,x_{\mathrm{im}}$represent covariates and $z_{i}=\left\{z_{\mathrm{il}},\ldots ,z_{\mathrm{ip}}\right\}$a set of SNPs. The SNP-set kernel association tests for the null hypothesis that $h\left(z_{\mathrm{il}},\ldots ,z_{\mathrm{ip}}\right)=0$in the semiparametric model:

(1) $$\log itP\left(yi=1\right)=\beta_{0}+\beta_{l}x_{\mathrm{im}}+h\left(z_{i}\right)\text{,}$$

where *y*_*i*_ is the phenotype, β_0_ is the intercept, $\beta_{1},\ldots ,\beta_{m}$ are the coefficients of the covariates, and *h* is a nonparametric function. Under certain constraints, the function *h* can be defined as for $h\left(z_{1}\right)=\cdot \sum_{i'=1}^{n}\cdot y_{t'}K\left(Z_{i,}Z_{i'}\right)$ some coefficients $Y_{i},\ldots ,Y_{n}$and a positive semi-definite kernel function *K*(⋅,⋅) by the representer theorem [31,32]. The 3 kernel functions are mathematically described in Additional file 1: Table S1.

### Minimum detectable relative risk

With ancestry-specific cohort sizes of approximately 220 subjects and an approximate 30% ALI incidence, we calculated 80% power to detect minimum detectable relative risks in the range of 1.6 - 2.0 within each ancestry-defined cohort [33]. We assumed an additive genetic model framework to test for haplotype differences at an alpha level of 0.05. Our cohorts could achieve smaller detectable effect sizes for more common haplotypes [33].

## Results

### Characteristics of study population

Of 474 subjects who were genotyped, MDS identified two major clusters corresponding to European (EA) and African (AA) ancestry. Twenty-eight individuals were identified as outliers and were excluded from subsequent analyses, leaving populations of 222 AA subjects and 224 EA subjects (Additional file 1: Table S2). Clinical characteristics are displayed in Table 1 and Figure 1A. In each population, the incidence of ALI was approximately 30%. Within each ancestry after excluding outliers, MDS analysis did not reveal persistent population stratification.

**Table 1:** Study population and clinical covariates

**Variable**	**ALI (n = 142)**	**Non-ALI (n = 325)**	***p *-value**
Age, years	39 ± 19	37 ± 18	0.35
Male, n (%)	115 (81%)	249 (77%)	0.30
African ancestry, n (%)	61 (43%)	161 (48%)	0.29
European ancestry, n (%)	74 (51%)	150 (43%)	0.14
Era of injury 1999 – 2003, n (%)	85 (60%)	160 (49%)	0.07
**Injury Factors**			
Blunt trauma, n (%)	101 (71%)	218 (67%)	0.39
ISS	26 ± 8	24 ± 7	0.008
APACHE III †	64 ± 24	58 ± 18	0.004
Pulmonary contusion, n (%)	54 (38%)	76 (24%)	0.0013
**Treatment Factors**			
Total PRBC 1^st^ 24 h, units (Range)	3.16 (0 – 19)	1.56 (0 – 27)	<0.001
Mechanical ventilation, n (%)	142 (100%)	238 (73%)	<0.001
**Outcomes**			
Mortality, n (%)	35 (25%)	24 (7%)	<0.001
Hospital length of stay, days	22 (11 – 36)	13 (8 – 26)	<0.001

Most continuous variables are presented as mean ± standard deviation, and categorical variables are shown as number (n) and percentage of total population. Hospital length of stay is displayed as median (interquartile range) due to a skewed distribution. *ISS: *injury severity scale; *APACHE III *†: Acute physiology and chronic health evaluation, modified to omit the arterial blood gas oxygenation criterion given its collinearity with ALI; *PRBC: *packed red blood cell transfusion. Blunt trauma is opposed to penetrating trauma. Statistics shown reflect the results of binomial testing, Student’s *T *test, or nonparametric testing (Wilcoxon rank sum) as appropriate given the data distributions.

### Identification of ALI candidate genes

Twenty-two genes with previous published associations with ALI were identified in 2 review articles from 2008 and 2009 [3,5]. Between 2009 and May 2011, our search criteria returned 7 additional genetic associations with ALI or ALI outcome [13,15,16,34-41]. Of these 29 genes, 4 were not covered by the IBC chip, leaving 25 genes for this analysis (Table 2 and Figure 1B). For each prior ALI-associated gene, Table 2 lists population details about the original association study or studies, including ALI risk factor and population ancestry.

**Table 2:** Candidate Gene list

**Candidate Gene (gene symbol)**	**# SNPs AA**	**# SNPs EA**	**ALI Risk Factor**	**Ancestry**	**Ref**
Angiotensin converting enzyme (ACE)	39	24	Mixed ICU	Eur	3,5
Angiopoietin-2 (ANGPT2)	63	52	Trauma	Afr, Eur	13, 35
Chemokine (CXC motif) ligand 2 (CXCL2)	5	4	Sepsis	Eur	3,5
Epidermal growth factor (EGF)	36	20	Mixed ICU; Sepsis	Eur	34
Factor V (F5)	84	77	Mixed ICU	Eur	3,5
FAS (FAS, TNF receptor superfamily, member 6)	27	18	Mixed ICU; SIRS	Eur	16
Interleukin-6 (IL6)	15	10	Mixed ICU; sepsis	Eur	3,5,40
Interleukin-8 (IL8)	9	7	Trauma	Eur	3,5
Interleukin-10 (IL10)	5	9	Mixed ICU; trauma	Eur	3,5,38
Interleukin-1 receptor associated kinase 3 (IRAK3)	25	22	Sepsis	Eur	14
Mannose binding lectin (MBL2)	7	9	Mixed ICU	Eur, Chi	3,5
Macrophage migration inhibitory factor (MIF)	1	1	Sepsis	Eur, Afr	3,5
Myosin light chain kinase (MYLK)	10	8	Sepsis; trauma	Eur, Afr	3,5
NAD(P)H dehydrogenase, quinone 1 (NQO1)	14	11	Trauma	Eur	3
Nuclear factor of kappa light polypeptide gene enhancer in B-cells 1 (NFKB1)	57	54	Mixed ICU	Eur	3,5
Nuclear factor of kappa light polypeptide gene enhancer in B-cells inhibitor, alpha (NFKBIA)	14	18	Mixed ICU	Eur	3,5,39
Nuclear factor (erythroid-derived 2-like) 2 (NFE2L2)	19	16	Trauma	Eur	3,5
Pre-B-cell colony enhancing factor (PBEF1)	7	5	Sepsis; mixed ICU	Eur	3,5
Plasminogen activator, urokinase (PLAU)	2	2	Sepsis; pneumonia	Eur	3,5
Plasminogen activator inhibitor type 1 (SERPINE1)	14	8	Pneumonia	Multi	15
Superoxide dismutase 3, extracellular (SOD3)	5	1	Sepsis; pneumonia	Eur	3,5
Toll-like receptor 1 (TLR1)	14	12	Sepsis	Eur, Afr	37
Tumor necrosis factor alpha (TNF)	3	3	Mixed ICU	Eur	3,5
Tumor necrosis factor beta, lymphotoxin alpha (LTA)	4	4	Mixed ICU	Eur	3,5
Vascular endothelial growth factor (VEGFA)	10	8	Mixed ICU	Eur	3,5,41
**Genes not covered by IBC platform**					
Ferritin light polypeptide (FTL)	--	--	Mixed ICU	Eur	3,5
Heme oxygenase 2 (HMOX2)	--	--	Mixed ICU	Eur	3,5
Peptidase inhibitor 3, elafin (PI3)	--	--	Mixed ICU	Eur	5
Surfactant protein B (SFTPB)	--	--	Pneumonia, mixed ICU	Multi	3,5

Genetic variants with previous publications supporting an association with acute lung injury or acute respiratory distress syndrome are listed, along with the number of single nucleotide polymorphisms (SNPs) on the IBC platform. The genetic coverage varies for African (AA) or European ancestry (EA) because some variants are exclusive to one population. The IBC platform was designed to capture approximately 80% of the genetic variation for each gene within a cosmopolitan, or multi-ethnic, population [21]. The one exception to this extent of coverage among ALI candidate genes was for the myosin light chain kinase gene (MYLK), a very large gene, for which the genomic coverage was approximately 50% of known variation [21]. For each ALI candidate gene, characteristics of the original study population(s) are also listed, including ALI risk factor and population ancestry. *SIRS:* systemic inflammatory response syndrome; *Eur:* European ancestry; *Afr:* African ancestry; *Chi:* Chinese ancestry; *Multi:* multi-ethnic ancestry, not analyzed independently by ethnicity. *Ref:* reference citation.

### Genotyping and filtering

After filtering the raw genotyping data for polymorphic SNPs with call rates > 95%, Hardy-Weinberg equilibrium p-value > 10^-4^, and minor allele frequency > 0.05, 30,064 informative SNPs remained for EA subjects and 35,977 informative SNPs remained for AA subjects (Additional file 1: Table S3).

We grouped SNPs that survived filtering by annotation to ALI candidate genes, and 25 of 29 genes had 2 or more informative SNPs. Using the IBC chip data, European and African ancestry haplotype blocks were constructed in Haploview for each ALI candidate gene. Table 2 depicts the genes interrogated and the number of SNPs per gene. After filtering, we tested 489 SNPs in AA subjects and 403 SNPs in EA subjects (Table 2). The number of SNPs varies between ancestries because some variants are private to only one ancestry [42]. Given the distinct linkage disequilibrium (LD) structure for each ancestry, the haplotype blocks vary between the two populations.

### SNP-set kernel association test

The results of the SKAT using linear, IBS, and quadratic kernels testing the association between ALI susceptibility and candidate gene haplotype blocks or overall candidate genes are presented in Table 3 and Table 4. Only haplotype blocks demonstrating significance with ALI (p-value < 0.05) are listed; all others were non-significant. In the AA population, haplotype blocks in 4 candidate genes – interleukin (IL)6, IL10, interleukin-1 receptor associated kinase 3 (IRAK3), and vascular endothelial growth factor (VEGFA) – demonstrated a significant association with ALI. Each function of the kernel method also detected a strong association for angiopoietin- 2 (ANGPT2), which was previously reported for this population at the single SNP and haplotype level [13].

**Table 3:** Gene and haplotype ALI associations in African Ancestry trauma subjects

**Gene**	**N**	**SNPs**	**Single SNP p-value**	**Linear p-value**	**IBS p-value**	**Quadratic p-value**
IL6 block 1	219	3	0.018	0.021	0.057	0.0038
IL10 block 2	219	7	0.10	0.181	0.083	0.021
IRAK3 block 1	216	15	0.0048	0.070	0.044	0.16
VEGFA block 1	218	6	0.0035	0.77	0.032	0.056
*ANGPT2 block 2†*	*207*	*12*	*2.54E-05*	*4.5E-05*	*3.4E-05*	*1.0E-06*

In subjects of African ancestry, kernel machine regression detected significant ALI association for 4 ALI candidate genes: IL6, IL10, IRAK3, and VEGFA. The results of each of 3 kernel functions – linear, identity by state (IBS), and quadratic – are listed, and are contrasted with the most extreme individual SNP result for the block in question. Individual SNP results for the genes IRAK3 and VEGFA demonstrated a more extreme association with ALI than the haplotype block as tested by kernel function. However, the individual SNPs detected were not the same SNPs as have been previously associated with ALI [14,41]. In addition, results of kernel regression are reported for ANGPT2(†); single SNP and haplotype association of ANGPT2 with ALI has previously been reported for this population [13]. We include these results to demonstrate that the kernel method remains effective in the presence of strong individual SNP results. *N: *number of subjects with complete data; *SNPs: *number of SNPs per ALI-associated block.

**Table 4:** Gene and haplotype ALI associations in European Ancestry trauma subjects

**Gene**	**N**	**SNPs**	**Single SNP p-value**	**Linear p-value**	**IBS p-value**	**Quadratic p-value**
NFKBIA block 1	211	4	0.012	0.051	0.077	0.042
VEGFA block 2	211	3	0.016	0.020	0.045	0.0088

Two ALI candidate gene haplotype blocks were significant among EA subjects, in genes NFKBIA and VEGFA. As in Table 3, the results of 3 different kernel functions are shown, and are contrasted with the individual SNP showing the strongest ALI association for the block in question. While the most extreme NFKBIA individual SNP association with ALI was stronger than that detected by kernel function testing, the individual SNP was distinct from ALI – associated variants previously reported for this gene [39]. *N:* number of subjects with complete data; *SNPs: *number of SNPs per ALI-associated block.

**Figure 2:**
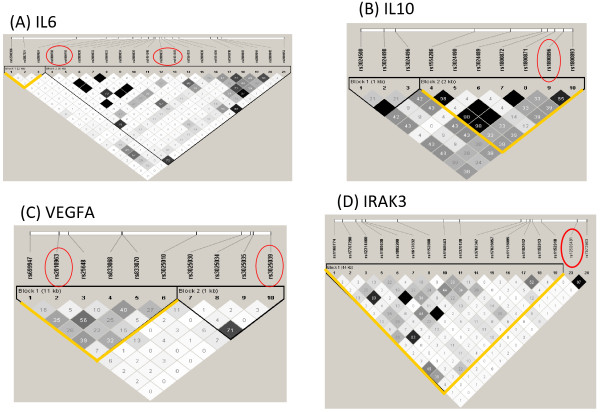
**Linkage disequilibrium (LD) plots for African ancestry (AA) trauma subjects for 4 genes with positive ALI associations.** Previously reported ALI-associated SNPs are circled in red, whereas the haplotype block detected to be ALI – associated by one or more kernel function is outlined in yellow. The grayscale reflects the pairwise LD as measured by r^2^, with high LD in black and low/absent LD in white. The ALI – associated genes are (**A**) IL6, (**B**) IL10, (**C**) VEGFA, and (**D**) IRAK3.

Haplotype blocks in 2 genes, nuclear factor of kappa light polypeptide gene enhancer in B-cells inhibitor, alpha (NFKBIA) and VEGFA, were associated with ALI among EA trauma subjects. Only VEGFA was common to both ancestries, and the ALI – associated block differed between them. The linkage disequilibrium (LD) plot and haplotype definitions for each ALI-associated gene are shown in Figures 2 (AA population) and 3 (EA population), with previously ALI-associated SNPs or regions circled. As illustrated by *VEGFA *, there is greater diversity in the AA population, which results in typically smaller haplotype blocks and a lesser degree of LD between block members.

**Figure 3:**
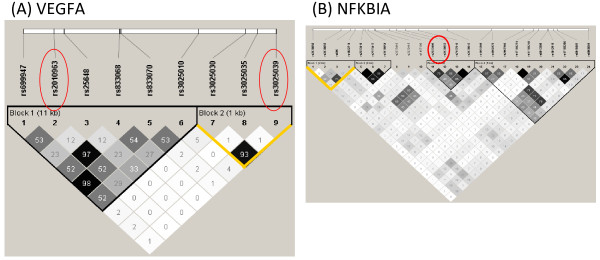
**LD plots for European ancestry (AA) trauma subjects for 2 genes with positive ALI associations.** Plots for the genes VEGFA (**A**) and NFKBIA (**B**) are shown. Grayscale reflects the pairwise LD as measured by r^2^, with high LD in black and low/absent LD in white. As in Figure 2, previously reported ALI – associated SNPs are circled (red) and the yellow lines outline the haplotypes detected by kernel function(s) as being associated with ALI. Whereas the block detected in VEGFA is defined by the previously reported SNP rs3025039, the association with NFKBIA does not overlap the previously reported NFKBIA ALI – associated locus.

Tables 3 and 4 also provide summary results of the most extreme single-SNP association with ALI for each SNP-set (haplotype block), assuming an additive model of genetic risk. In most cases, the kernel-based p-values demonstrate more extreme associations with ALI, since they draw information from multiple SNPs in the set.

### Comparison of kernel functions by matrix plots

**Figure 4:**
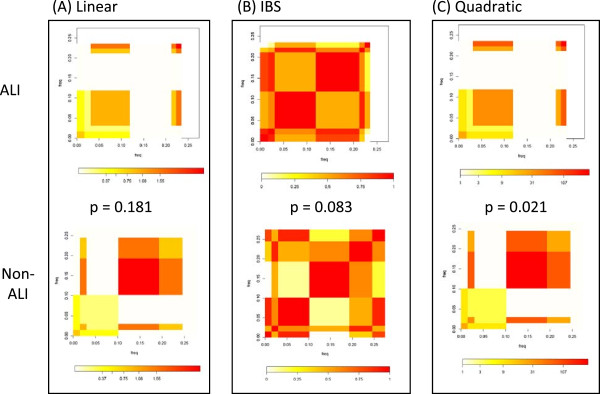
**Representative kernel matrix plot of the IL10 gene in AA subjects.** For each matrix, the ALI or non-ALI population of haplotypes is plotted against itself according to the kernel function. Each cell on the matrix reflects the extent of haplotype sharing between ALI subjects or non-ALI subjects. The cell size reflects the frequency of the haplotype, such that common haploypes are larger rectangles. The color reflects the kernel function value for each haplotype among ALI or non-ALI; more intense red coloration reflects a higher degree of similarity between pairs of subjects based on their diploid genotypes. Comparing the top and bottom plots in each box, one observes that the ALI (top) plots are distinct for each kernel function, but the plots are most discordant for the quadratic function, which allows for 2-way interactions between SNPs within the haplotype block to inform the association. Accordingly, the *p- *value for the quadratic kernel test with ALI was the most statistically significant.

**Figure 5:**
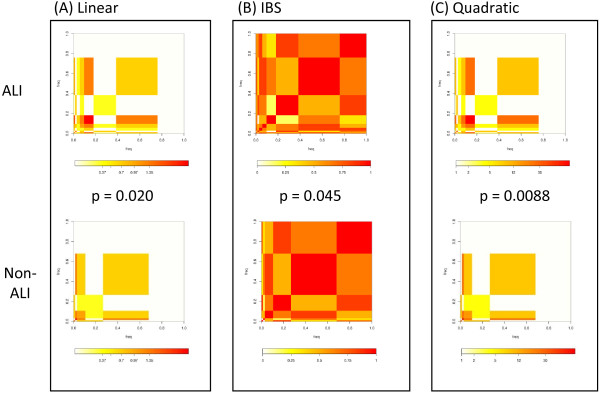
**Representative kernel matrix plot of the VEGFA gene in EA subjects.** As in Figure 4, the plot displays the frequency of haplotype sharing within ALI cases or non – cases, where size of the cell reflects the overall haplotype frequency and the red coloration reflects the degree of similarity among group members. In the case of VEGFA, both the coloration and block structure of ALI cases are most distinct with the quadratic function, and this is reflected in the most extreme *p- *value for the quadratic kernel test.

To visualize the genotype differences in cases and non-cases, we draw kernel matrix plots of paired haplotypes in Figures 4 and 5. The matrix plot thus summarizes the population genetic and kernel weight information for ALI and non-ALI subjects, providing an intuitive comparison of genetic similarity between the ALI and non-ALI populations. When the plots for ALI and non-ALI subjects appear similar, the candidate gene’s SNP-set kernel function does not vary significantly by ALI status, whereas distinct patterns for the ALI plot reflect an enrichment or paucity of specific haplotypes among ALI subjects.

Taking the example of IL10 haplotype block 2 in the AA population, the ALI plots become noticeably more distinct from the non-ALI as one progresses from Figures 4A (linear kernel) to 4B (IBS kernel) to 4C (quadratic kernel). Similarly, the kernel test *p* values become more extreme, achieving significance only for the quadratic kernel function. In the case of haplotype block 2 for VEGFA in EA subjects, the quadratic kernel matrix plot is more distinct for ALI subjects (Figure 5A**–**5C), complementing the results of Table 4 which indicate a more significant association for this block applying the quadratic kernel function. In the case of ANGPT2 hapBlock 2, the matrix plots are very distinct to ALI cases regardless of the kernel function applied (Additional file 2: Figure S1 and Additional file 3: Figure S2). By examining multiple kernel functions for each SNP-set, one can examine the relative contribution of rare allele sharing, SNP-by-SNP interaction, and strong SNP main effects to the association.

## Discussion

Replication is lacking for many ALI candidate genes, particularly among non-European populations. We leveraged information from large-scale genotyping to confirm associations with ALI at the gene level, and used haplotype blocks to refine the genomic association between gene and phenotype. We investigated 25 previously published genetic ALI associations in a cohort of trauma – associated ALI informative for both European and African ancestry, and found associations that were not readily apparent on an individual SNP-based analysis, replicating associations for IL6, IL10, IRAK3, VEGFA, and NFKBIA. To our knowledge, we are only the second group to report a large scale genetic replication study in ALI; the prior study used genome wide data in a European ancestry case – control population and replicated only 2 prior ALI-associated SNPs [43]. As the field of ALI genetics matures, it will be important to continue to attempt replication in ALI of different inciting causes, in multiple ethnicities, and across multiple genotyping platforms [22]. Our cohort was uniquely poised to attempt replication in that we had moderate throughput, dense genotyping of over 2000 high-priority genes; the population followed a cohort design, with prospective enrollment of critically ill subjects at significant risk for developing ALI; the population was equally divided between Americans of European and African ancestry, allowing modest power for each ancestry; and all cohort members shared the same risk factor for ALI.

By grouping SNPs into haplotypes or genes, the SNP-set kernel association test reduces the dimensionality of the problem and allows for interaction between members of the SNP-set while still adjusting for covariate effects. As each kernel function arrives at a different assessment of similarity between individuals and assigns a different weight to the possible joint function of SNPs within a SNP set, the SNP-set *p* value varies between linear, IBS, and quadratic models for the same SNP-set. In most cases, the results are similar between models, although different patterns emerge in Tables 3 and 4. When the *p-*values are more extreme for the quadratic function compared to the IBS or linear kernel as is the case for IL6 block 1 in AAs or VEGFA block 2 in EAs, it suggests that the SNPs in the associated haplotype may associate with the phenotype predominantly through significant interaction terms, since this kernel incorporates 2-way interactions and quadratic main effects. In contrast, the IBS kernel measures the number of minor alleles shared among individuals, suggesting that when the haplotype block associates strongly with ALI by the IBS kernel, minor allele states contribute heavily to the association. This may be the case for VEGFA block 1 (AA).

We introduce a novel graphical representation of the kernel results with our matrix plots. The equivalence of kernel machine method, genomic distance-based regression, and haplotype dissimilarity test has been understood theoretically [44,45]. In case–control data, the test in genomic distance-based regression can be simplified to a contrast of haplotype compositions between cases and controls [44]. Our matrix plot provides a new way to visualize such contrast, providing additional insights to understand whether the genotype frequency, LD structure, or the kernel function plays the important role in reaching a significant conclusion.

SNP-set analysis seems to be a more powerful method to replicate findings across different populations and different platforms. For genes IL6, IL10, and NFKBIA, the previously reported individual SNPs were not significant at even the nominal additive level (Additional file 1: Table S4). Only VEGFA rs3025039 (C/T + 936) demonstrated marginal association with ALI, with a p-value of 0.018 in Europeans. As such, a traditional application of individual-SNP associations with ALI would have failed to detect replication for any candidate SNPs in the AA population, and would missed a replication with NFKBIA in Europeans. Furthermore, the fact that in several instances, the haplotype detected by SNP-set analysis matched that containing the previously ALI - associated SNP lends support to the robustness of the replicated association.

In AA subjects, positive associations were confirmed for IL6, IL10, IRAK3, and VEGFA. For each gene, this is the first replication with ALI susceptibility in a non-European population. Interleukin-6 is the best replicated genetic association with ALI, with 4 previous reported associations [3,5,40]. Ours is the first report of an association specific to trauma-associated ALI, suggesting that IL6 variation is a critical genetic factor across multiple forms of ALI. Both a functional IL6 promoter SNP (−174 G/C, rs1800795G) and a gene-wide haplotype are ALI risk factors in Europeans. In our population, the association was strongest for IL6 haplotype block 1 rather than for the block containing rs1800795. Similar to rs1800795, block 1 is in the upstream promoter region of IL6, though in vivo or *in vitro* data about its effect on IL6 expression in African American subjects are not available. Interestingly, rs1800795G, the European risk allele, is the dominant allele in African and African American populations, with allele frequency > 90% [42]. Our results suggest that haplotype variation in block 1 strongly influences ALI susceptibility even with minimal variation at the rs1800795 locus. The quadratic kernel function returned the most extreme statistical association, suggesting that 2-way interactions between SNPs in IL6 haplotype block 1 may strongly inform the association.

For IL10, the quadratic function yielded a significant association for block 2, with members demonstrating modest LD (r^2^ = 0.40) to the previously reported ALI – associated IL10 promoter SNP rs1800896 [38], despite single-SNP results which showed no apparent association. The current study represents the 2^nd^ positive association between IL10 variation and trauma-associated ALI; the gene has also been implicated as an ALI risk factor in a predominantly septic mixed ICU population [38,43]. There has been a suggestion that the effect of IL10 promoter variation may be modified by clinical factors including age, and that it may also modify an individual’s risk for mortality once ALI is established [38]. Within our AA subjects, IL10 variation remained significantly associated with ALI after adjusting for age and severity of illness, among other clinical factors. The current study further extends the relevance of this gene into non-European subjects at risk for ALI.

The association of IRAK3 with ALI in African trauma subjects is interesting in that this block shows essentially no LD (r^2^ < 0.10) with rs10506481, the SNP previously reported to associate with ALI in a Spanish population and which was in LD with a putative transcription factor binding site disrupting SNP among Spanish subjects [14]. With the present study, IRAK3 variation gains traction as a risk factor generalizable to non-infectious ALI, as well as to African populations. Previous reports have demonstrated a variable degree of North African admixture in various Spanish populations [46], although the degree of African admixture within the study population was not reported for the previous IRAK3 study [14]. Further mechanistic studies will be necessary to determine whether the IL6, IL10, and IRAK3 associations reported here represent the same functionality as those described previously.

Among European ancestry trauma subjects, our report is the first to replicate NFKBIA and represents the third report of an association between VEGFA and ALI. Haplotype block 1 of NFKBIA is in the downstream region of the gene and does not show strong LD with the previously reported promoter SNPs (rs3138053, rs2233406, rs2233409) associated with ALI, which reside in block 3 of this gene [39]. As the previous study genotyped only the promoter SNPs, it remains uncertain whether the kernel – detected block reflects a novel association for this gene. One of the previously reported promoter SNPs (rs2233409C) typed by the IBC platform demonstrated marginal association with ALI on the individual SNP level (p = 0.064, Table S3). Ours is the first generalization of NFKBIA as a risk factor specific to trauma-associated ALI. Previously, haplotypes in the gene associated with an increased risk of ALI in a mixed ICU population, the majority of whom had sepsis. It remains unknown whether NFKBIA haplotype variation results in altered gene or protein expression of inhibitor I κB, which binds the transcription factor NF-κB and prevents its translocation to the nucleus.

For VEGFA, among European ancestry subjects haplotype block 2 is defined by rs3025039, a SNP previously reported to associate with ALI mortality [41]. Of note, VEGFA was the only gene detected by SKAT as significant in both ancestries, and while the block identified in AA subjects was distinct from that in EA subjects, both blocks are defined by previously reported ALI – associated variants. The possible interaction of ancestry and VEGFA structure on ALI susceptibility is an interesting consideration. Ours is the first report of an association between ALI and VEGFA in non-Europeans, and the first report of VEGFA as a trauma-specific risk factor. With our report, VEGFA rivals IL6 as the most replicated ALI genetic risk factor, and focuses attention on the critical contribution of endothelial dysfunction to the development of ALI [47,48].

Our study has several important limitations. While we are encouraged by the replicated associations with relatively modest ancestry-specific populations of just over 200 subjects each, we were underpowered to detect SNP-level genetic relative risks (GRR) below 1.5, and it is likely that for a complex trait such as ALI, GRR < 1.5 are common [49]. As such, our study’s failure to replicate previous associations must be interpreted with caution. This may be particularly relevant for genes previously implicated as influencing trauma-associated ALI, including IL8, MYLK, NQO1, and NFE2L2. However, failure to replicate in our gene-based analysis could also represent a fundamental difference between the genetic regulators of trauma-associated ALI compared to ALI incited by sepsis or pneumonia, which are the populations best studied for ALI susceptibility. While the leukocyte gene expression pattern of subjects with severe blunt trauma was remarkably similar to that of subjects exposed to low dose endotoxin [50], there may be substantial differences between patients with infection and those with injury that result in different mechanisms leading to ALI as a shared phenotype.

Failure to replicate an association could also be due to the gene being inadequately genotyped by the IBC chip (e.g., surfactant protein B), or when the previously reported variant was a structural variant rather than a SNP, as in the case of the angiotensin converting enzyme (ACE) or plasminogen activating inhibitor 1 (PAI1) [3,5,15]. The IBC SNP array was not designed to capture structural variation such as insertion/deletion (I/D) polymorphisms, and our genotyping did not disclose I/D or copy number status of structural genetic variants. In addition, one limitation of the SNP-set kernel association test as opposed to the traditional SNP-based tests of association is that the kernel function returns a two-tailed score statistic calculated under the null hypothesis, as opposed to an effect estimate such as the odds ratio. Thus, while the SKAT is able to detect multiple interactions, including those between SNPs within an LD block acting in opposing directions on the risk for ALI, the SKAT does not reveal which haplotype is overrepresented in ALI and which is underrepresented. Rather, it focuses attention on the ALI-associated haplotype block and prompts further haplotype analysis to obtain an estimate of the odds ratio.

Genetic risk for ALI warrants closer examination in non-European populations. By using a gene-based method to detect ALI association, we have been able to extend the number of ALI-associated genes specific to AA populations from 4 to 8. We know even less about the genetic influences on ALI in Asian or admixed populations, and future cohorts should be designed specific to these populations. It remains possible that the inherited response to injury varies widely between ancestries [51], and it may be that different genetic structures within the same gene exert a more dominant effect in different populations. Our results for VEGFA may be a particular example in this regard. It will be critically important to understand population differences in candidate gene regulation if the findings from genetic studies are to be successfully translated into personalized therapeutic options in the future.

## Conclusions

Replication is lacking for many ALI candidate genes, particularly among non-European populations. Using a kernel machine regression methodology based on haplotype – defined SNP sets, we confirmed ALI associations with IL6, IL10, IRAK3, VEGFA for the first time among African American trauma subjects. We have also extended the relevance of VEGFA and NFKBIA as specific to trauma-associated ALI among European Americans. By refining the genetic association signals and the host populations most likely to demonstrate specific genetic risks for ALI susceptibility or outcome, we may be better positioned to design individualized preventative or therapeutic options for future ALI at-risk populations.

## Competing interests

The authors declare that they have no competing interests.

## Authors’ contributions

Study conception and design: NJM, PNL, RA, MGS, JDC, and RF. Data acquisition, analysis, and interpretation: NJM, ZJD, MR, and RF. Drafting and editing the manuscript: NJM, ZJD, RA, MGS, PNL, JDC, and RF. All authors read and approved the final manuscript.

## Pre-publication history

The pre-publication history for this paper can be accessed here:

http://www.biomedcentral.com/1471-2350/13/52/prepub

## Supplementary Material

Additional file 1 **Supplementary Tables. **SNP-Set Analysis Replicates Acute Lung Injury Genetic Risk Factors. Click here for file

Additional file 2 **Figure S1. **ANGPT AA population haplotype blocks. Block 2, highlighted in yellow, was associated with ALI by all 3 kernel function regressions (Linear IBS, and quadratic). In addition, this block contains the 2SNPs previously reported to associate with trauma-associated ALI by Meyer et al. 2011 [13]. Click here for file

Additional file 3 **Figure S2. **ANGPT2 block 2, kernel matrix plots. The ALI population has a distinct kernel representation regardless of the kernel function applied, reflected by significant *p *values for each kernel methodology. Click here for file
