# Supplementary material for: SNP-set analysis replicates acute lung injury genetic risk factors
Source: BMC Med Genet. 2012 Jun 28;13:52. doi: 10.1186/1471-2350-13-52 (PMC3512475; doi:10.1186/1471-2350-13-52)
Supplement: Additional file 1 — Supplementary Tables. SNP-Set Analysis Replicates Acute Lung Injury Genetic Risk Factors. [file 1471-2350-13-52-S1.doc]

| **Variable** | **ALI (n=142)** | **Non-ALI (n=325)** | ***p*-value** |
| --- | --- | --- | --- |
| Age, years | 39 ± 19 | 37 ± 18 | 0.35 |
| Male, n (%) | 115 (81%) | 249 (77%) | 0.30 |
| African ancestry, n (%) | 61 (43%) | 161 (48%) | 0.29 |
| European ancestry, n (%) | 74 (51%) | 150 (43%) | 0.14 |
| Era of injury 1999 – 2003, n (%) | 85 (60%) | 160 (49%) | 0.07 |
| **Injury Factors** | | | |
| Blunt trauma, n (%) | 101 (71%) | 218 (67%) | 0.39 |
| ISS | 26 ± 8 | 24 ± 7 | 0.008 |
| APACHE III † | 64 ± 24 | 58 ± 18 | 0.004 |
| Pulmonary contusion, n (%) | 54 (38%) | 76 (24%) | 0.0013 |
| **Treatment Factors** | | | |
| Total PRBC 1^st^ 24 hrs, units (Range) | 3.16 (0 – 19) | 1.56 (0 – 27) | < 0.001 |
| Mechanical ventilation, n (%) | 142 (100%) | 238 (73%) | < 0.001 |
| **Outcomes** | | | |
| Mortality, n (%) | 35 (25%) | 24 (7%) | <0.001 |
| Hospital length of stay, days | 22 (11 – 36) | 13 (8 – 26) | <0.001 |

**Table 1: Study population and clinical covariates.**

Most continuous variables are presented as mean ± standard deviation, and categorical variables are shown as number (n) and percentage of total population. Hospital length of stay is displayed as median (interquartile range) due to a skewed distribution. *ISS:* injury severity scale; *APACHE III* †: Acute physiology and chronic health evaluation, modified to omit the arterial blood gas oxygenation criterion given its collinearity with ALI; *PRBC:* packed red blood cell transfusion. Blunt trauma is opposed to penetrating trauma. Statistics shown reflect the results of binomial testing, Student’s T test, or nonparametric testing (Wilcoxon rank sum) as appropriate given the data distributions.

| **Candidate Gene (gene symbol)** | **# SNPs AA** | **# SNPs EA** | **ALI Risk Factor** | **Ancestry** | **Ref** |
| --- | --- | --- | --- | --- | --- |
| Angiotensin converting enzyme (ACE) | 39 | 24 | Mixed ICU | Eur | 3,5 |
| Angiopoietin-2 (ANGPT2) | 63 | 52 | Trauma | Afr, Eur | 13, 35 |
| Chemokine (CXC motif) ligand 2 (CXCL2) | 5 | 4 | Sepsis | Eur | 3,5 |
| Epidermal growth factor (EGF) | 36 | 20 | Mixed ICU; Sepsis | Eur | 34 |
| Factor V (F5) | 84 | 77 | Mixed ICU | Eur | 3,5 |
| FAS (FAS, TNF receptor superfamily, member 6) | 27 | 18 | Mixed ICU; SIRS | Eur | 16 |
| Interleukin-6 (IL6) | 15 | 10 | Mixed ICU; sepsis | Eur | 3,5,44 |
| Interleukin-8 (IL8) | 9 | 7 | Trauma | Eur | 3,5 |
| Interleukin-10 (IL10) | 5 | 9 | Mixed ICU; trauma | Eur | 3,5,45 |
| Interleukin-1 receptor associated kinase 3 (IRAK3) | 25 | 22 | Sepsis | Eur | 14 |
| Mannose binding lectin (MBL2) | 7 | 9 | Mixed ICU | Eur, Chi | 3,5 |
| Macrophage migration inhibitory factor (MIF) | 1 | 1 | Sepsis | Eur, Afr | 3,5 |
| Myosin light chain kinase (MYLK) | 10 | 8 | Sepsis; trauma | Eur, Afr | 3,5 |
| NAD(P)H dehydrogenase, quinone 1 (NQO1) | 14 | 11 | Trauma | Eur | 3 |
| Nuclear factor of kappa light polypeptide gene enhancer in B-cells 1 (NFKB1) | 57 | 54 | Mixed ICU | Eur | 3,5 |
| Nuclear factor of kappa light polypeptide gene enhancer in B-cells inhibitor, alpha (NFKBIA) | 14 | 18 | Mixed ICU | Eur | 3,5,47 |
| Nuclear factor (erythroid-derived 2-like) 2 (NFE2L2) | 19 | 16 | Trauma | Eur | 3,5 |
| Pre-B-cell colony enhancing factor (PBEF1) | 7 | 5 | Sepsis; mixed ICU | Eur | 3,5 |
| Plasminogen activator, urokinase (PLAU) | 2 | 2 | Sepsis; pneumonia | Eur | 3,5 |
| Plasminogen activator inhibitor type 1 (SERPINE1) | 14 | 8 | Pneumonia | Multi | 15 |
| Superoxide dismutase 3, extracellular (SOD3) | 5 | 1 | Sepsis; pneumonia | Eur | 3,5 |
| Toll-like receptor 1 (TLR1) | 14 | 12 | Sepsis | Eur, Afr | 37 |
| Tumor necrosis factor alpha (TNF) | 3 | 3 | Mixed ICU | Eur | 3,5 |
| Tumor necrosis factor beta, lymphotoxin alpha (LTA) | 4 | 4 | Mixed ICU | Eur | 3,5 |
| Vascular endothelial growth factor (VEGFA) | 10 | 8 | Mixed ICU | Eur | 3,5,48 |
| **Genes not covered by IBC platform** |  |  |  | |  |
| Ferritin light polypeptide (FTL) | -- | -- | Mixed ICU | Eur | 3,5 |
| Heme oxygenase 2 (HMOX2) | -- | -- | Mixed ICU | Eur | 3,5 |
| Peptidase inhibitor 3, elafin (PI3) | -- | -- | Mixed ICU | Eur | 5 |
| Surfactant protein B (SFTPB) | -- | -- | Pneumonia, mixed ICU | Multi | 3,5 |

**Table 2: Candidate Gene list.** Genetic variants with previous publications supporting an association with acute lung injury or acute respiratory distress syndrome are listed, along with the number of single nucleotide polymorphisms (SNPs) on the IBC platform. The genetic coverage varies for African (AA) or European ancestry (EA) because some variants are exclusive to one population. The IBC platform was designed to capture approximately 80% of the genetic variation for each gene within a cosmopolitan, or multi-ethnic, population (21). The one exception to this extent of coverage among ALI candidate genes was for the myosin light chain kinase gene (MYLK), a very large gene, for which the genomic coverage was approximately 50% of known variation (21). For each ALI candidate gene, characteristics of the original study population(s) are also listed, including ALI risk factor and population ancestry. *SIRS:* systemic inflammatory response syndrome; *Eur:* European ancestry; *Afr:* African ancestry; *Chi:* Chinese ancestry; *Multi:* multi-ethnic ancestry, not analyzed independently by ethnicity. *Ref:* reference citation.

| Gene | N | SNPs | Single SNP p-value | Linear p-value | IBS p-value | Quadratic p-value |
| --- | --- | --- | --- | --- | --- | --- |
| IL6 block 1 | 219 | 3 | 0.018 | 0.021 | 0.057 | 0.0038 |
| IL10 block 2 | 219 | 7 | 0.10 | 0.181 | 0.083 | 0.021 |
| IRAK3 block 1 | 216 | 15 | 0.0048 | 0.070 | 0.044 | 0.16 |
| VEGFA block 1 | 218 | 6 | 0.0035 | 0.77 | 0.032 | 0.056 |
| *ANGPT2 block 2†* | *207* | *12* | *2.54E-05* | *4.5E-05* | *3.4E-05* | *1.0E-06* |

**Table 3: Gene and haplotype ALI associations in African Ancestry trauma subjects.**

In subjects of African ancestry, kernel machine regression detected significant ALI association for 4 ALI candidate genes: IL6, IL10, IRAK3, and VEGFA. The results of each of 3 kernel functions – linear, identity by state (IBS), and quadratic – are listed, and are contrasted with the most extreme individual SNP result for the block in question. Individual SNP results for the genes IRAK3 and VEGFA demonstrated a more extreme association with ALI than the haplotype block as tested by kernel function. However, the individual SNPs detected were not the same SNPs as have been previously associated with ALI (14, 45). In addition, results of kernel regression are reported for ANGPT2(†); single SNP and haplotype association of ANGPT2 with ALI has previously been reported for this population (13). We include these results to demonstrate that the kernel method remains effective in the presence of strong individual SNP results.

| Gene | N | SNPs | Single SNP p-value | Linear  p-value | IBS p-value | Quadratic p-value |
| --- | --- | --- | --- | --- | --- | --- |
| NFKBIA block 1 | 211 | 4 | 0.012 | 0.051 | 0.077 | 0.042 |
| VEGFA block 2 | 211 | 3 | 0.016 | 0.020 | 0.045 | 0.0088 |

**Table 4: Gene and haplotype ALI associations in European Ancestry trauma subjects.** Two ALI candidate gene haplotype blocks were significant among EA subjects, in genes NFKBIA and VEGFA. As in Table 3, the results of 3 different kernel functions are shown, and are contrasted with the individual SNP showing the strongest ALI association for the block in question. While the most extreme NFKBIA individual SNP association with ALI was stronger than that detected by kernel function testing, the individual SNP was distinct from ALI – associated variants previously reported for this gene (47).
