# Supplementary material for: SNP-set analysis replicates acute lung injury genetic risk factors
Source: BMC Med Genet. 2012 Jun 28;13:52. doi: 10.1186/1471-2350-13-52 (PMC3512475; doi:10.1186/1471-2350-13-52)
Supplement: Additional file 2 — Figure S1. ANGPT AA population haplotype blocks. Block 2, highlighted in yellow, was associated with ALI by all 3 kernel function regressions (Linear IBS, and quadratic). In addition, this block contains the 2SNPs previously reported to associate with trauma-associated ALI by Meyer et al. 2011 [13]. [file 1471-2350-13-52-S2.ppt]

## Slide 1
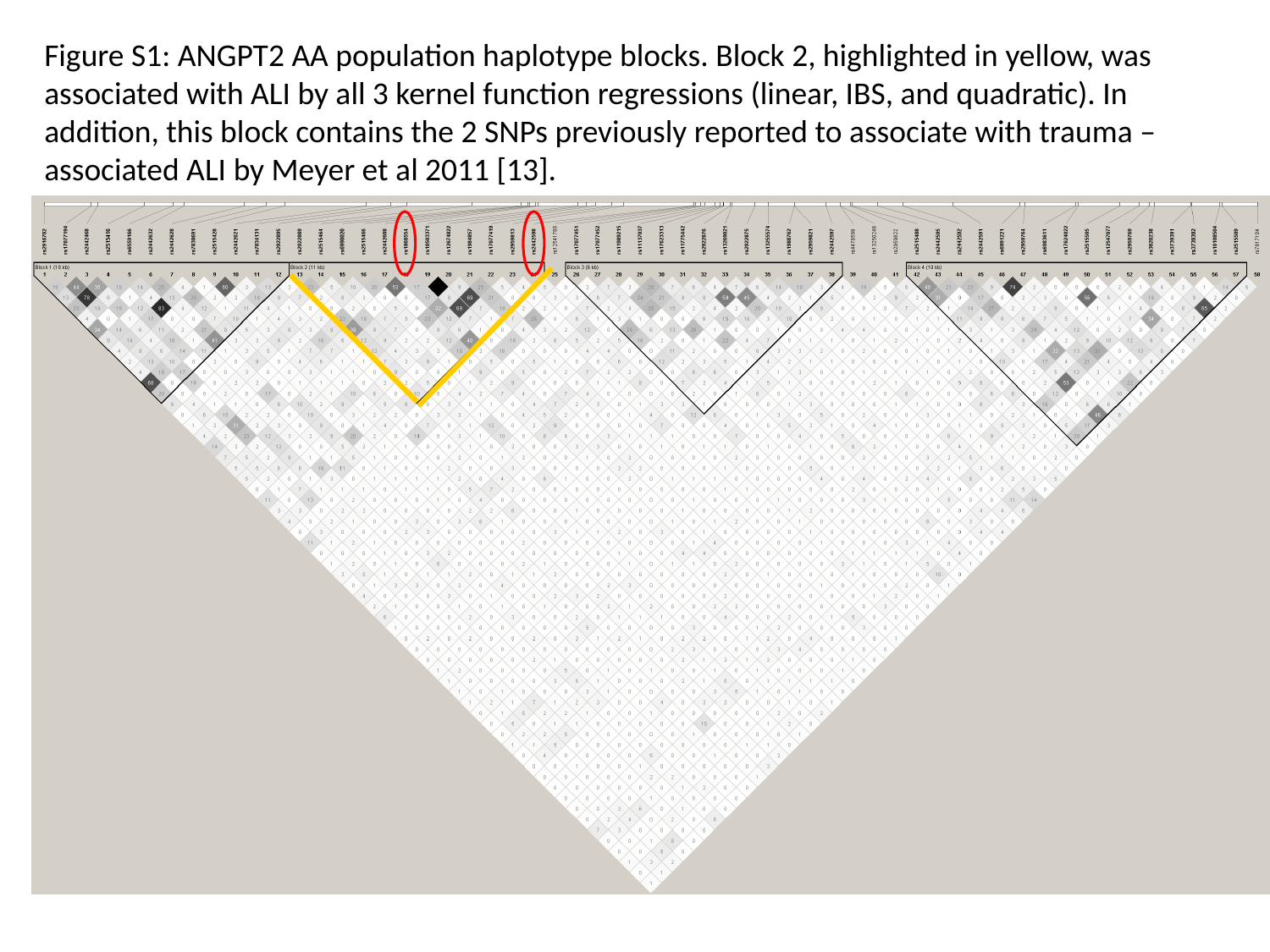

# Figure S1: ANGPT2 AA population haplotype blocks. Block 2, highlighted in yellow, was associated with ALI by all 3 kernel function regressions (linear, IBS, and quadratic). In addition, this block contains the 2 SNPs previously reported to associate with trauma – associated ALI by Meyer et al 2011 [13].
