# Supplementary material for: SNP-set analysis replicates acute lung injury genetic risk factors
Source: BMC Med Genet. 2012 Jun 28;13:52. doi: 10.1186/1471-2350-13-52 (PMC3512475; doi:10.1186/1471-2350-13-52)
Supplement: Additional file 3 — Figure S2. ANGPT2 block 2, kernel matrix plots. The ALI population has a distinct kernel representation regardless of the kernel function applied, reflected by significant p values for each kernel methodology. [file 1471-2350-13-52-S3.ppt]

## Slide 1
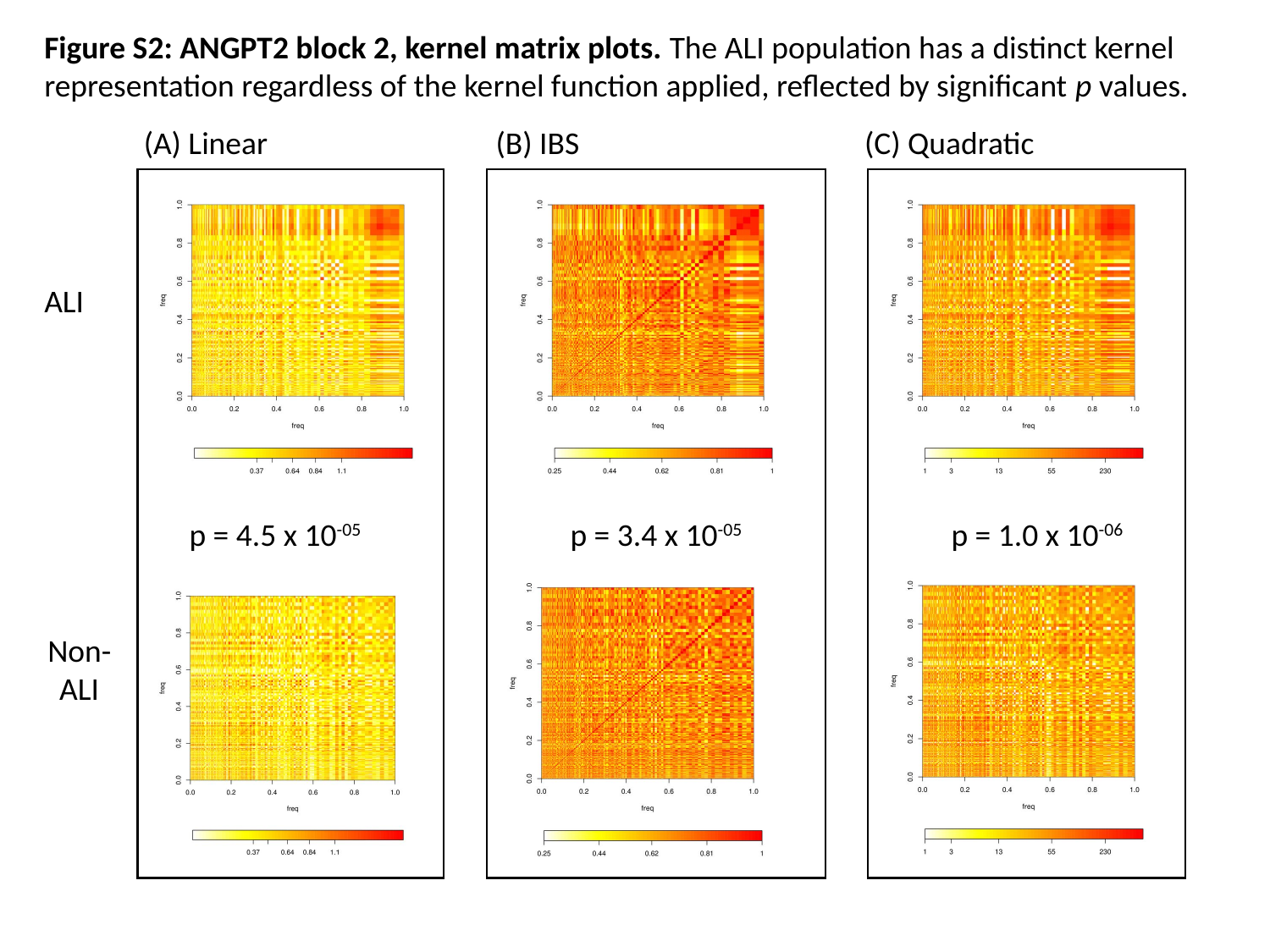

Figure S2: ANGPT2 block 2, kernel matrix plots. The ALI population has a distinct kernel representation regardless of the kernel function applied, reflected by significant p values.
 (A) Linear		 (B) IBS		 (C) Quadratic
ALI
p = 4.5 x 10^-05^
p = 3.4 x 10^-05^
p = 1.0 x 10^-06^
Non- ALI
